# Supplementary material for: SDS-22 stabilizes GSP-1/-2 PP1 subunits contributing to polarity establishment in C. elegans embryos
Source: EMBO Rep. 2025 Nov 6;26(24):6240–65. doi: 10.1038/s44319-025-00624-0 (PMC12714725; doi:10.1038/s44319-025-00624-0)
Supplement: Supplementary file 1 — Appendix [file 44319_2025_624_MOESM1_ESM.pdf]

# Table of Contents

# Appendix Figures S1-S7 and Figure legends


Appendix Figure S1: Volcano plot of proteins identified in the GSP-2 immunoprecipitation. pg. 2

Appendix Figure S2: The E153A substitution does not result in reduced levels of SDS-22. pg. 3

Appendix Figure S3: SDS-22, GSP-1 and GSP-2 were depleted in co-depletion experiments. pg. 4

Appendix Figure S4: SDS-22 levels in GSP-1 and/or GSP-2 depleted embryos. pg. 5

Appendix Figure S5: Depletion of RPN-6.1 shows the same effect as RPN-7 depletion. pg. 6-7

Appendix Figure S6: Depletion of SDS-22::GFP is efficient in the co-depletion experiments. pg. 8

Appendix Figure S7: Depletion of SDS-22 from L1 larval stage results in reduction of GFP::GSP-1 intensity comparable to SDS-22(E153A) mutant embryos. pg. 9

**Li et al., Appendix Figure S1**

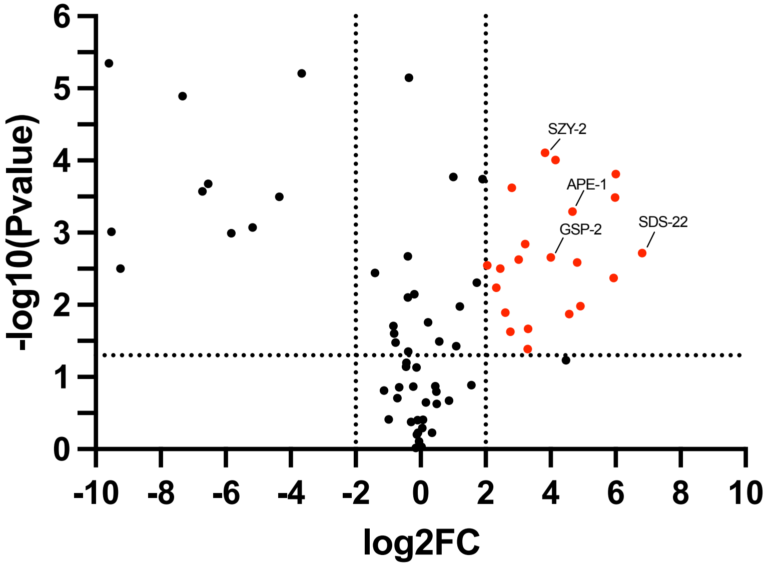


**Volcano plot of proteins identified in the GSP-2 immunoprecipitation.**
Volcano plot showing the GSP-2 interactors. The proteins highlighted are the identified regulators of GSP-2 using a threshold of log2FC≥2 (dotted lines on the log2FC≥2 axis). The Y axis is the -log10 (P value) and the dotted line represent a P value < 0.05.

# Li et al., Appendix Figure S2

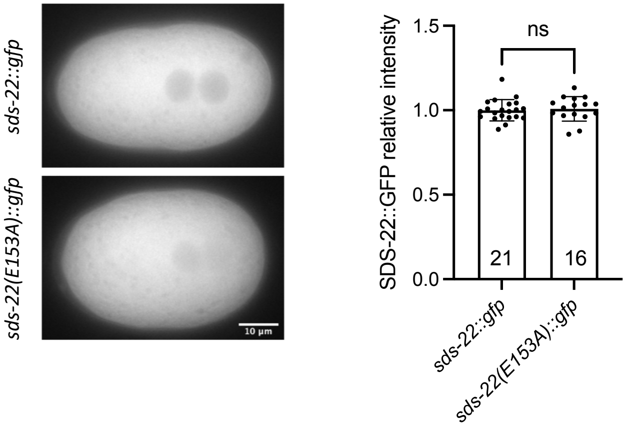


**The E153A substitution does not result in reduced levels of SDS-22.**
Images of *sds-22::gfp* and *sds-22(E153A)::gfp* at pronuclear meeting stage, left and quantification of relative levels of SDS-22(E153A)::GFP normalized to SDS-22::GFP levels, right. For all embryos, the scale bar is 10 µm, anterior is to the left and posterior to the right. Mean is shown and error bars indicate SD. Each dot represents a single embryo. Sample size (*n*) is indicated inside the bars in the graph. *N* = 2. ns p > 0.05. The P-values were determined using two-tailed unpaired Student's t-test. Exact P-values are provided in Dataset EV3. *n* = number of embryos analyzed; *N* = number of independent experiments.

# Li et al., Appendix Figure S3

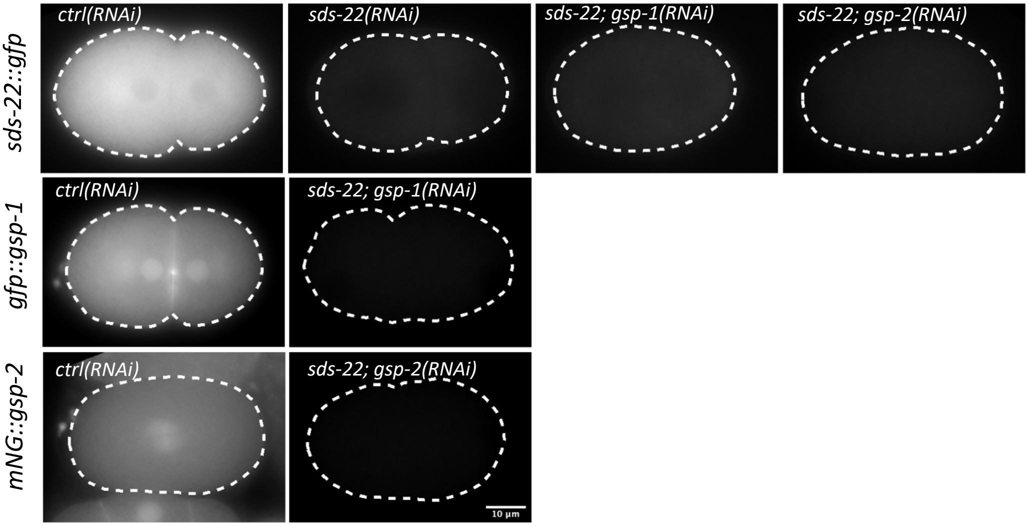


**SDS-22, GSP-1 and GSP-2 were depleted in co-depletion experiments.**
Representative images of the indicated genotypes. Co-depletion of SDS-22 with GSP-1 or GSP-2 is efficient. For all embryos, the scale bar is 10 µm, anterior is to the left and posterior to the right. RNA interference was performed by injection.

# Li et al., Appendix Figure S4

A

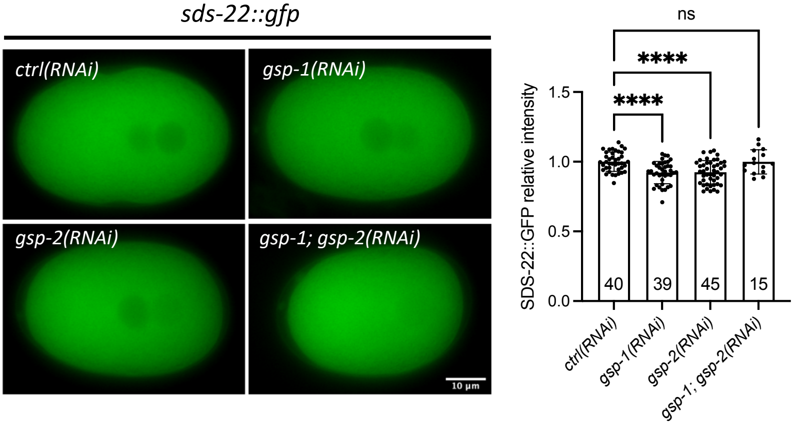


B

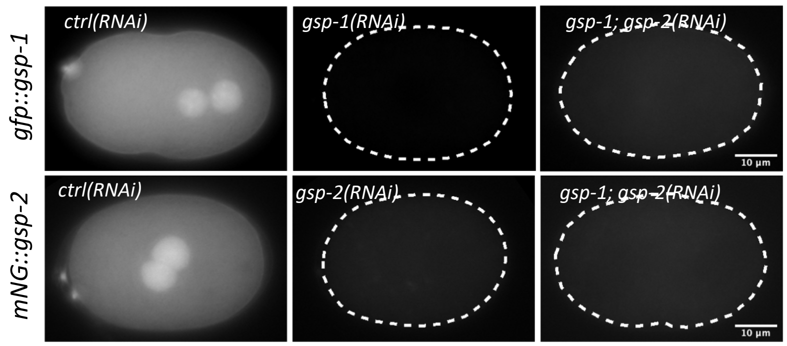


**SDS-22 levels in GSP-1 and/or GSP-2 depleted embryos.**

**(A)** Left, representative images of *sds-22::gfp* embryos in *ctrl(RNAi), gsp-1(RNAi), gsp-2(RNAi)* and *gsp-1(RNAi); gsp-2(RNAi).* Right, quantification of relative SDS-22::GFP levels. Mean is shown and error bars indicate SD. Sample size (*n*) is indicated inside the bars in the graph. Each dot represents a single embryo. *N* = 3. ns $p > 0.05$, ****$p < 0.0001$. The P-values were determined using one-way ANOVA "Tukey's multiple comparisons test". Exact P-values are provided in Dataset EV3. *n* = number of embryos analyzed; *N* = number of independent experiments. **(B)** Representative images of the indicated genotypes. GSP-1 and GSP-2 are efficiently depleted in the indicated co-depletions. RNA interference was performed by injection. For all embryos, the scale bar is 10 µm, anterior is to the left and posterior to the right.

# Li et al., Appendix Figure S5

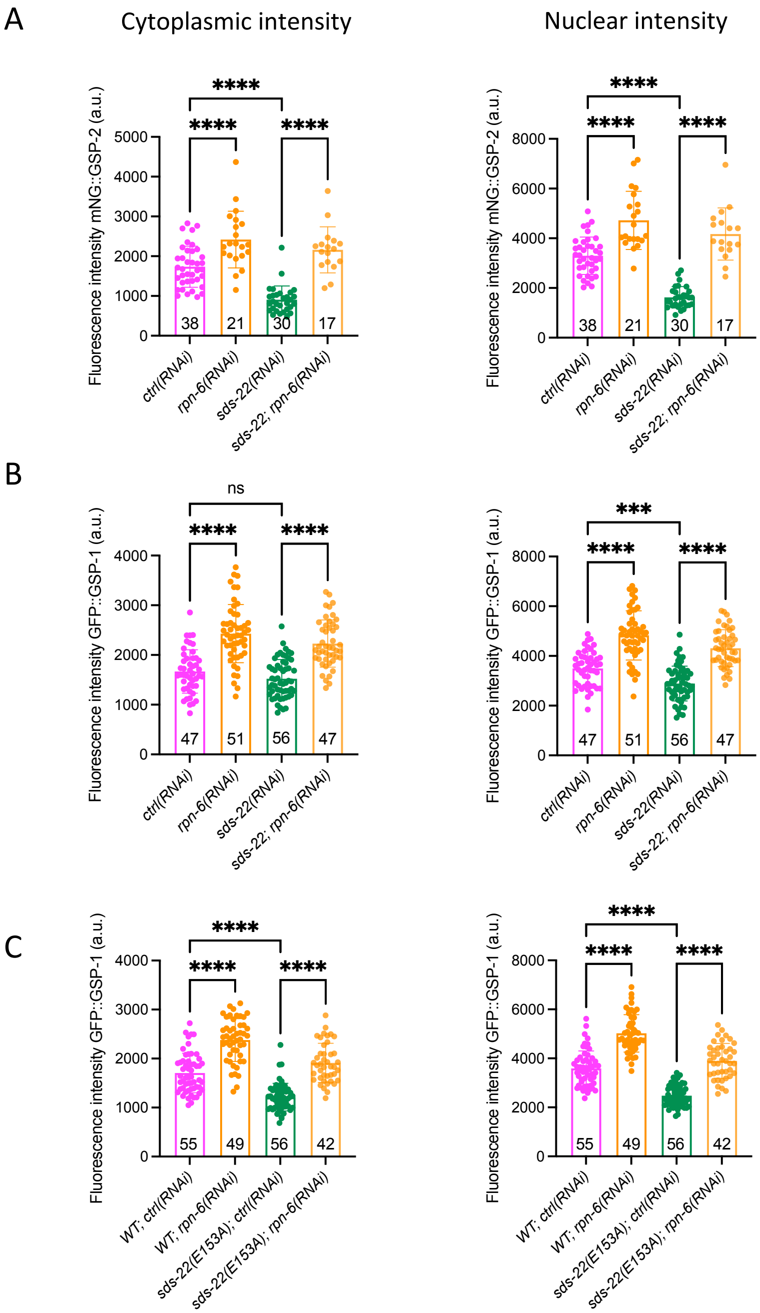

**Depletion of RPN-6.1 shows the same effect as RPN-7 depletion.**
**(A-B)** Quantification of mNG::GSP-2 **(A)** and GFP::GSP-1 **(B)** intensity levels in the cytoplasm and nucleus of -1 and -2 oocytes in the *ctrl(RNAi), sds-22(RNAi), rpn-6(RNAi),* and *sds-22(RNAi); rpn-6(RNAi)* conditions. *N* = 3. **(C)** Quantification of GFP::GSP-1 intensity levels in the cytoplasm and nucleus of -1 and -2 oocytes, comparing wild type, SDS-22(E153A) mutation, *ctrl(RNAi)* and *rpn-6(RNAi)*. *N* = 3. In all plots, mean is shown and error bars indicate SD. Each dot represents the measurement of one germline. Sample size (*n*) is indicated inside the bars in the graph. The RNP-6.1 has been performed in parallel to the RNP-7 depletion assay of Fig. 7, and it is therefore the same *ctrl(RNAi)* and *sds-22(RNAi)* data as shown in Fig. 7B, C, E, F, H and I. For all panels, RNA interference was performed by feeding. The scale bars are 50 µm. The P-values were determined using one-way ANOVA "Tukey's multiple comparisons test". ns $p > 0.05$, * $p < 0.05$, ** $p < 0.01$, ***$p < 0.001$, ****$p < 0.0001$. Exact P-values are provided in Dataset EV3. *n* = number of embryos analyzed; *N* = number of independent experiments.

# Li et al., Appendix Figure S6

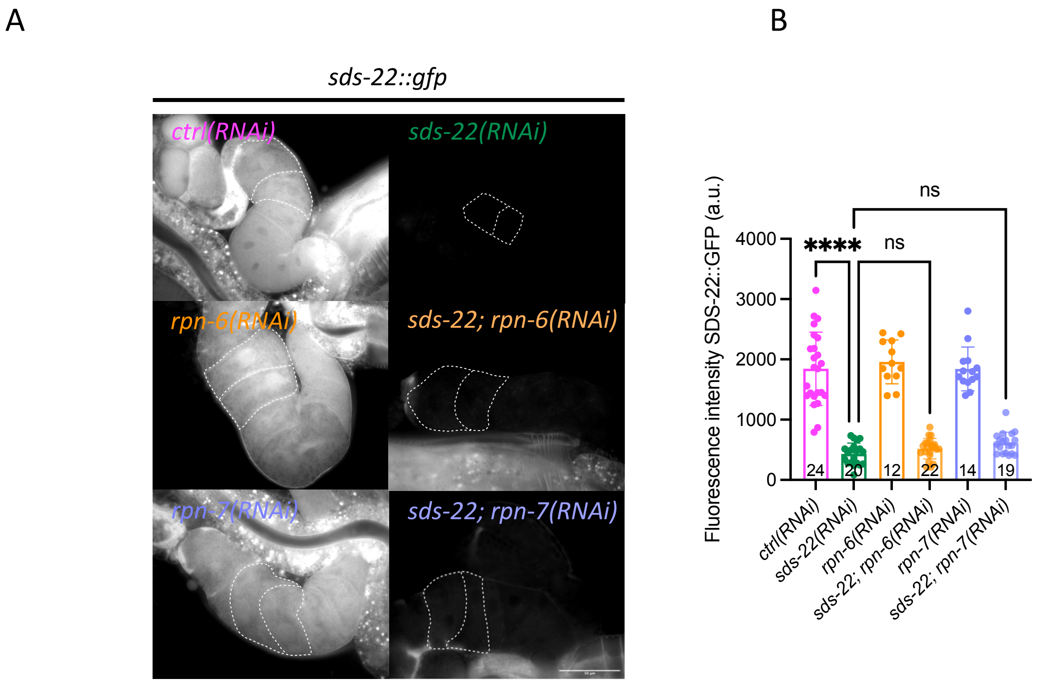


**Depletion of SDS-22::GFP is efficient in the co-depletion experiments.**
**(A)** Representative midsection images of *sds-22::gfp* germlines in *ctrl(RNAi), sds-22(RNAi), rpn-6(RNAi)* or *rpn-7(RNAi)* single depletion, and *sds-22(RNAi); rpn-6(RNAi)* and *sds-22(RNAi); rpn-7(RNAi)* co-depletion. No viable zygotes were produced after depletion of RPN-6/-7 alone or co-depletion with SDS-22, indicating that RPN-6 and RPN-7 were well depleted in these conditions. Scale bar is 50 µm. **(B)** Quantification of SDS-22::GFP intensity levels in the cytoplasm of -1 and -2 oocytes. Mean is shown and error bars indicate SD. Each dot represents the measurement of one germline. Sample size (*n*) is indicated inside the bars in the graph. *N* = 3. The P-values were determined using one-way ANOVA "Tukey's multiple comparisons test". ns $p > 0.05$, ****$p < 0.0001$. Exact P-values are provided in Dataset EV3 . *n* = number of embryos analyzed; *N* = number of independent experiments.

# Li et al., Appendix Figure S7

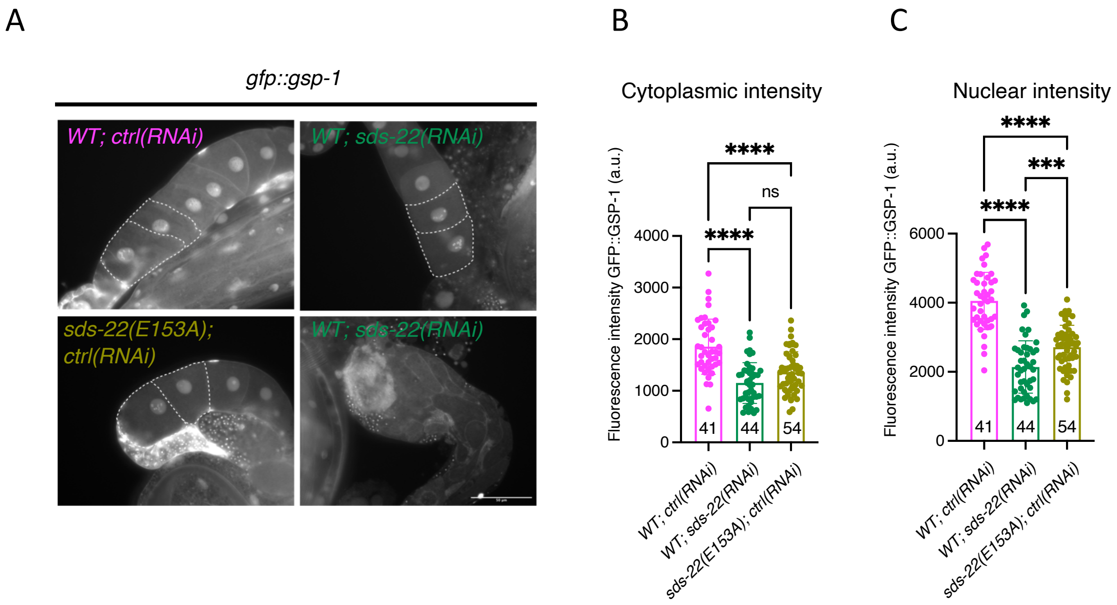


**Depletion of SDS-22 from L1 larval stage results in reduction of GFP::GSP-1 intensity comparable to SDS-22(E153A) mutant embryos.**
**(A)** Representative images of *gfp::gsp-1* germlines in *ctrl(RNAi)* and *sds-22(RNAi),* and *gfp::gsp-1;sds-22(E153A)* germlines in *ctrl(RNAi).* **(B, C)** Quantification of GFP::GSP-1 intensity levels in the cytoplasm **(B)** and nucleus **(C)** of -1 and -2 oocytes. $N = 3$. Mean is shown and error bars indicate SD. Each dot represents the measurement of one germline. The P-values were determined using one-way ANOVA "Tukey's multiple comparisons test". Sample size (*n*) is indicated inside the bars in the graph. For all panels, RNA interference was performed by feeding. The scale bars are 50 µm. In all plots, ns p > 0.05, * p < 0.05, ** p < 0.01, ***p < 0.001, ****p < 0.0001. Exact P-values are provided in Dataset EV3. *n* = number of embryos analyzed; *N* = number of independent experiments.
