# Supplementary material for: SDS-22 stabilizes GSP-1/-2 PP1 subunits contributing to polarity establishment in C. elegans embryos
Source: EMBO Rep. 2025 Nov 6;26(24):6240–65. doi: 10.1038/s44319-025-00624-0 (PMC12714725; doi:10.1038/s44319-025-00624-0)
Supplement: Supplementary file 6 — Movie EV1 [file 44319_2025_624_MOESM6_ESM.zip › EMBOR-2025-61928V2_Movie_EV1/Movie EV1_readme.docx]

**Movie EV1: *sds-22(RNAi)* rescues PAR-2 posterior cortical localization in the *pkc-3(ne4246); gfp::par-2* embryos.**

*gfp::par-2* and *pkc-3*(*ne4246*); *gfp::par-2* embryos treated either with *ctrl*(*RNAi*) or *sds-22*(*RNAi*) (RNAi by feeding). Acquisition of midplane fluorescent images begins during the early establishment phase, and frames are captured every 10 s. In *gfp::par-2* embryos (both *ctrl*(*RNAi*) and *sds-22*(*RNAi*)) PAR-2 localizes at the posterior cortex in the one-cell stage embryo and in P1 in the two-cell stage embryo (*n* = 12 for both). In *pkc-3*(*ne4246*); *gfp::par-2*, *ctrl*(*RNAi*) PAR-2 is uniformly distributed at the cortex in both one- and two-cell stage embryo (*n* = 42). After depletion of SDS-22, *pkc-3*(*ne4246*); *gfp::par-2* embryos showed PAR-2 posterior localization in one-cell stage embryo and in the P1 cell (*n* = 42 out of 48). *N* = 5. *n* = number of embryos analyzed; *N* = number of independent experiments. Anterior is to the left and posterior to the right.
