# Supplementary material for: SDS-22 stabilizes GSP-1/-2 PP1 subunits contributing to polarity establishment in C. elegans embryos
Source: EMBO Rep. 2025 Nov 6;26(24):6240–65. doi: 10.1038/s44319-025-00624-0 (PMC12714725; doi:10.1038/s44319-025-00624-0)
Supplement: Supplementary file 7 — Movie EV2 [file 44319_2025_624_MOESM7_ESM.zip › EMBOR-2025-61928V2_Movie_EV2/Movie EV2_readme.docx]

**Movie EV2: *sds-22*(*E153A*) substitution reduces the length of the PAR-2 cortical domain.**

Acquisition of midplane fluorescent images begins during the early stage, and frames are captured every 10 s. In *gfp::par-2; sds-22(E153A)* mutant embryos the length of cortical PAR-2 domain is decreased compared to *gfp::par-2* embryos at pronuclear meeting stage (*n* = 30 and *n* = 52, respectively). *N* = 3. *n* = number of embryos analyzed. *N* = number of independent experiments. Anterior is to the left and posterior to the right.  Referred to Fig 3A,B.
