# Supplementary material for: SDS-22 stabilizes GSP-1/-2 PP1 subunits contributing to polarity establishment in C. elegans embryos
Source: EMBO Rep. 2025 Nov 6;26(24):6240–65. doi: 10.1038/s44319-025-00624-0 (PMC12714725; doi:10.1038/s44319-025-00624-0)
Supplement: Supplementary file 8 — Movie EV3 [file 44319_2025_624_MOESM8_ESM.zip › EMBOR-2025-61928V2_Movie_EV3/Movie EV3_readme.docx]

**Movie EV3: *sds-22*(*E153A*) substitution rescues PAR-2 posterior cortical localization in the *pkc-3*(*ne4246*); *gfp::par-2* embryos at 22°C*.***

Acquisition of midplane fluorescent images begins during the early stage, and frames are captured every 10 s. In *gfp::par-2; pkc-3*(*ne4246*); *sds-22(E153A)* mutant embryos PAR-2 is more enriched to the posterior cortex compared to *gfp::par-2; pkc-3*(*ne4246*) embryos where PAR-2 is localized all over the cortex (*n* = 19 for both genotypes). *N* = 3. *n* = number of embryos analyzed. *N* = number of independent experiments. Anterior is to the left and posterior to the right.  Referred to Fig 3F,G.
