# Supplementary material for: SDS-22 stabilizes GSP-1/-2 PP1 subunits contributing to polarity establishment in C. elegans embryos
Source: EMBO Rep. 2025 Nov 6;26(24):6240–65. doi: 10.1038/s44319-025-00624-0 (PMC12714725; doi:10.1038/s44319-025-00624-0)
Supplement: Supplementary file 9 — Expanded View Figures [file 44319_2025_624_MOESM9_ESM.pdf]

Expanded View Figures

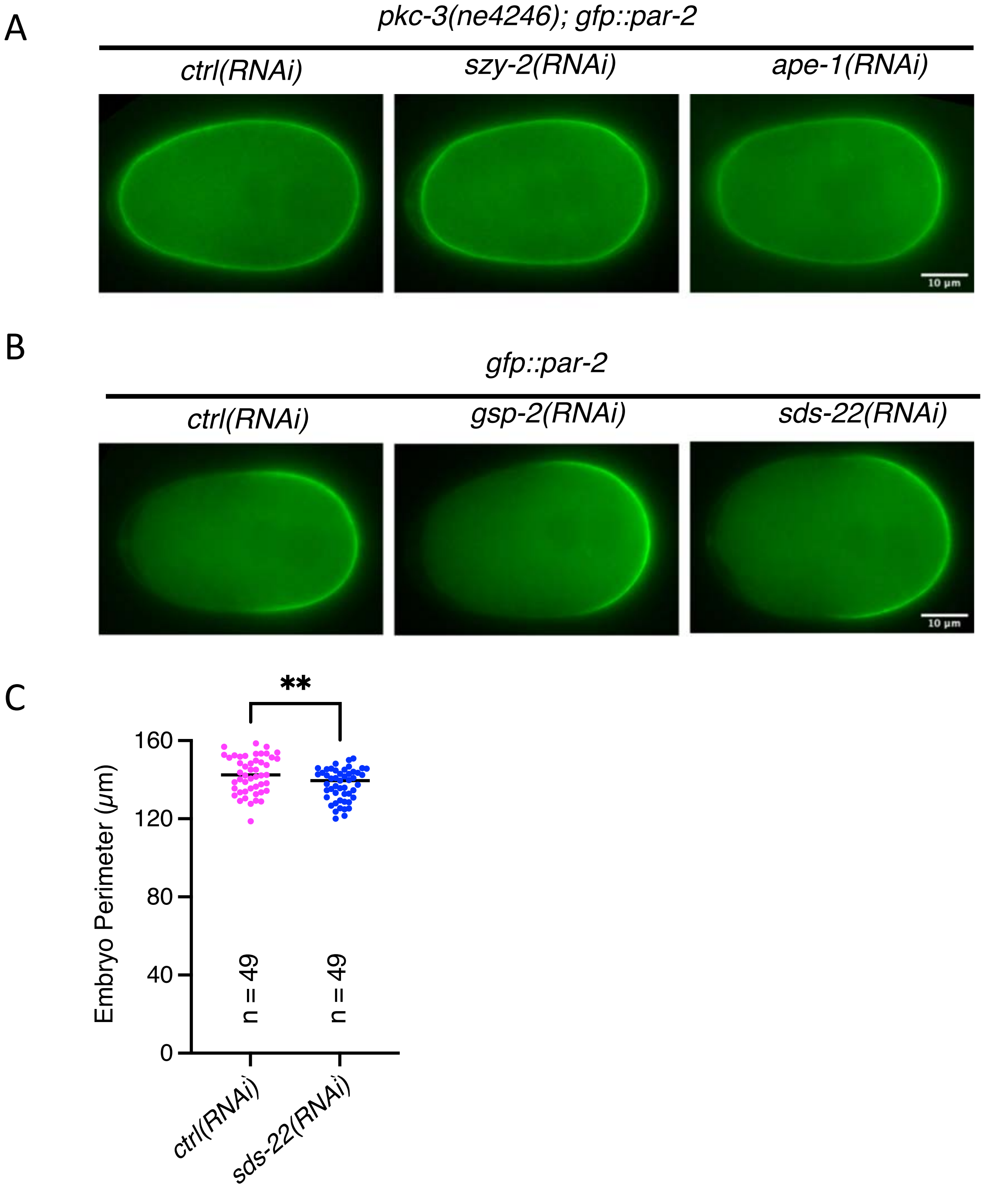

Figure EV1. PAR-2 localization after depletion in different genetic backgrounds of candidate regulators of PP1.

(**A**) Still images of embryos at the pronuclear meeting stage taken from time-lapse videos: *pkc-3(ne4246); gfp::par-2; ctrl(RNAi)*, $n = 18$, *szy-2(RNAi)*, $n = 17$, $N = 4$, and *ape-1(RNAi)*, $n = 16$, $N = 2$. (**B**) Still images of embryos at the pronuclear meeting stage taken from time-lapse videos of *gfp::par-2*, comparing *ctrl(RNAi)*, $n = 33$, *gsp-2(RNAi)*, $n = 24$ and *sds-22(RNAi)*, $n = 35$. $N = 3$. The quantification of the size of the PAR-2 domain is displayed in Fig. 1D. (**C**) Quantification of embryo perimeter with indicated RNAi conditions. For both *ctrl(RNAi)* and *sds-22(RNAi)*, $n = 49$, $N = 6$. For the images in (**B**) and quantifications of the perimeter in (**C**) we used a subset of the embryos of the experiment of Fig. 4. The *P* value was determined using two-tailed unpaired Student's *t* test. Mean is shown and each dot represents a single embryo. ns $P > 0.05$, *$P < 0.05$, **$P < 0.01$, ***$P < 0.001$, ****$P < 0.0001$. Exact *P* values are provided in Dataset EV3. RNA interference was performed by feeding. For all embryos, the scale bar is 10 µm, anterior is to the left and posterior to the right. *n* number of embryos analyzed, *N* number of independent experiments.

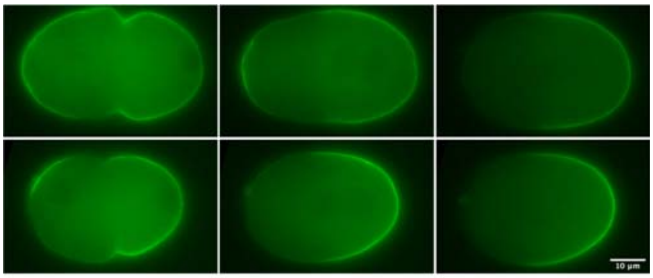

	Pronuclear migration	Pronuclear meeting	Mitosis
	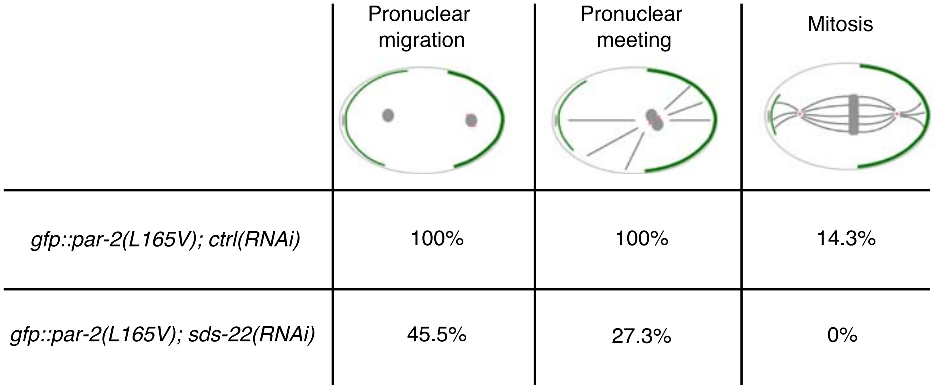		
gfp::par-2(L165V); ctrl(RNAi)	100%	100%	14.3%
gfp::par-2(L165V); sds-22(RNAi)	45.5%	27.3%	0%

Figure EV2. Depletion of SDS-22 rescues aberrant PAR-2 localization in *gfp::par-2(L165V)* mutant.

Top: still images from time lapse videos of *gfp::par-2(L165V)* of one-cell embryos at different cell division stages, *ctrl(RNAi)*, *n* = 9 and *sds-22(RNAi)*, *n* = 11, *N* = 3. For all embryos, the scale bar is 10 μm, anterior is to the left and posterior to the right. Bottom: the table shows the percentage of the phenotype represented schematically at the top. RNA interference was performed by feeding. *n* number of embryos analyzed, *N* number of independent experiments.

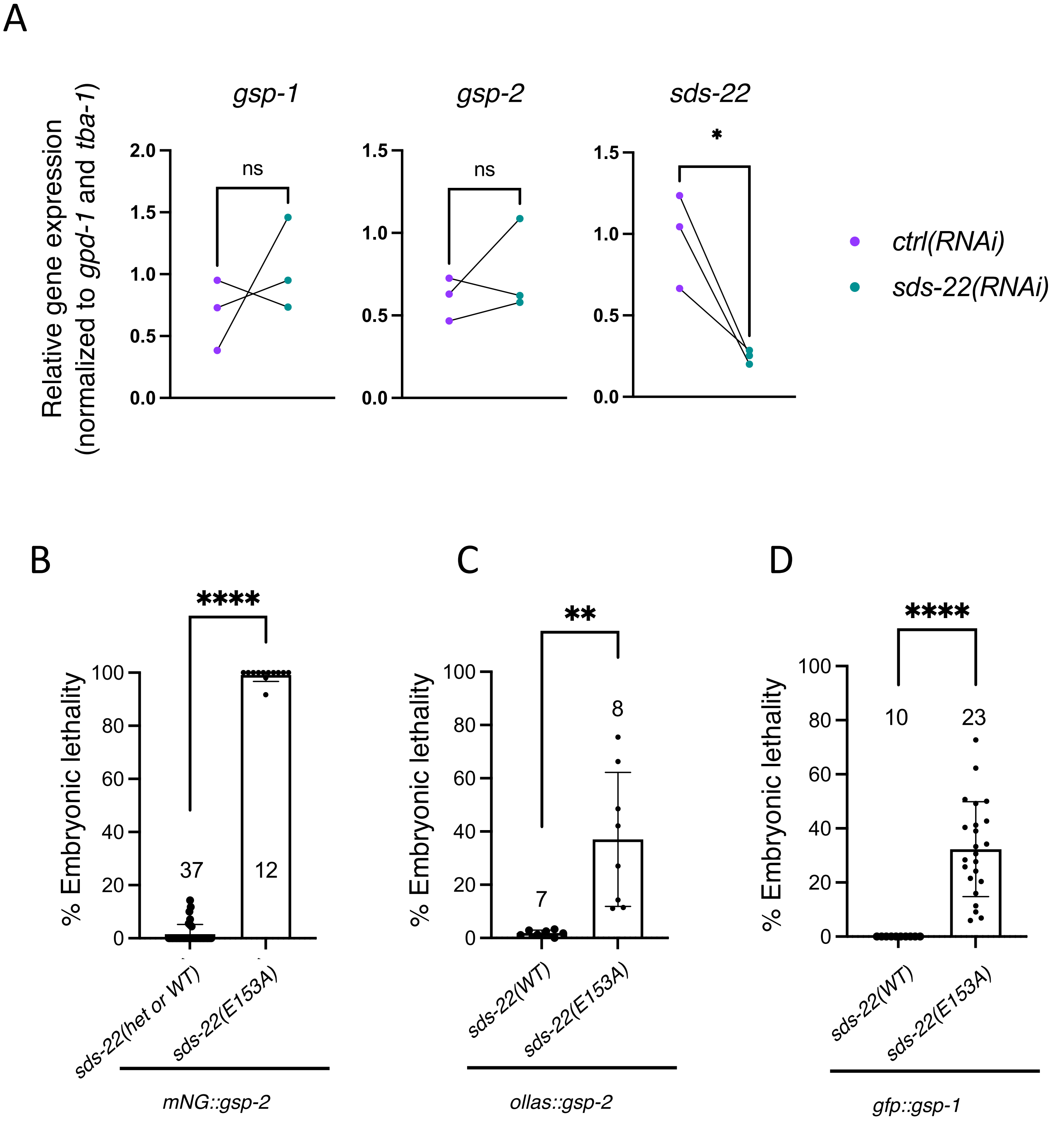

Figure EV3. The E153A substitution in SDS-22 leads to embryonic lethality in the *mNG::gsp-2, ollas::gsp-2* and *gfp::gsp-1* genetic backgrounds.

(**A**) Quantitative PCR of *gsp-1, gsp-2* and *sds-22* in *N2* following SDS-22 depletion. The geometrical mean between two control genes (*gpd-1* and *tba-1*) was used to calculate the relative gene expression. The statistical significance has been calculated by paired *t* tests comparing ΔCt values of different samples. Each dot is the value of one experiment. $N = 3$. RNA interference was performed by feeding. (**B–D**) Embryonic lethality of embryos with the *sds-22(E153A)* mutation in the genetic background of *mNG::gsp-2* (**B**), *ollas::gsp-2* (**C**) and *gfp::gsp-1* (**D**). The reported values correspond to the percentage of unhatched embryos over the total progeny (larvae and unhatched embryo). *mNG::gsp-2; sds-22(E153A/+ or +/+)*, $n = 1703$, *mNG::gsp-2; sds-22(E153A)*, $n = 568$. $N = 3$. *ollas::gsp-2*, $n = 476$, *ollas::gsp-2; sds-22(E153A)*, $n = 470$. $N = 2$. *gfp::gsp-1*, $n = 713$, *gfp::gsp-1; sds-22(E153A)*, $n = 1783$. $N = 2$. In this plot, each dot represents the quantified lethality of one single plate. Mean is shown and error bars indicate SD. The *P* values were determined using two-tailed unpaired Student's *t* test. In all plots, ns $P > 0.05$, $^*P < 0.05$, $^{**}P < 0.01$, $^{****}P < 0.0001$. Exact *P* values are provided in Dataset EV3. *n* number of embryos analyzed, *N* number of independent experiments.

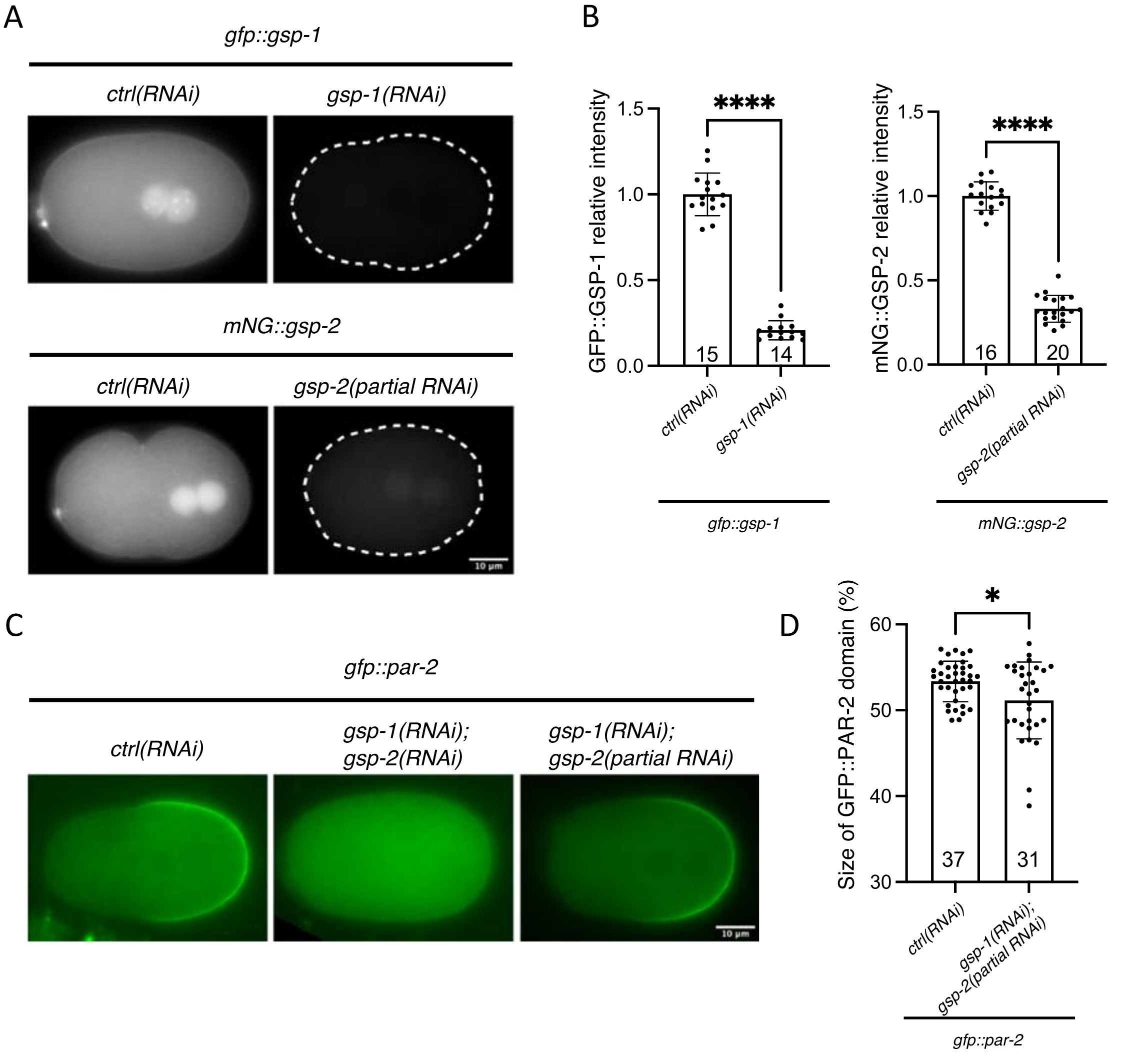

Figure EV4. PAR-2 polarizes with minimum amounts of GSP-1 and GSP-2.

(**A**) Upper, representative images of *gfp::gsp-1* embryos in *ctrl(RNAi)* and *gsp-1(RNAi)*. RNA interference was performed by injection. Lower, representative images of *mNG::gsp-2* embryos in *ctrl(RNAi)* and *gsp-2(RNAi)*. RNA interference was performed by feeding diluted bacteria to obtain a partial depletion. (**B**) Quantification of relative GFP::GSP-1 and mNG::GSP-2 levels. $N = 2$. (**C**) Representative images of *gfp::par-2* embryos with the indicated depletion. Co-depletion of GSP-1 and GSP-2 was performed by injection (middle panel) and by injection of GSP-1 dsRNA and feeding diluted GSP-2 RNAi bacteria (right panel). (**D**) Quantification of the GFP::PAR-2 domain size at pronuclear meeting in live zygotes. $N = 3$. In all plots, mean is shown and error bars indicate SD. Sample size (*n*) is indicated inside the bars in the graph, each dot represents a single embryo. *$P < 0.05$, ****$P < 0.0001$. The *P* values were determined using two-tailed unpaired Student's *t* test. Exact *P* values are provided in Dataset EV3. *n* number of embryos analyzed, *N* number of independent experiments.

 © The Author(s)

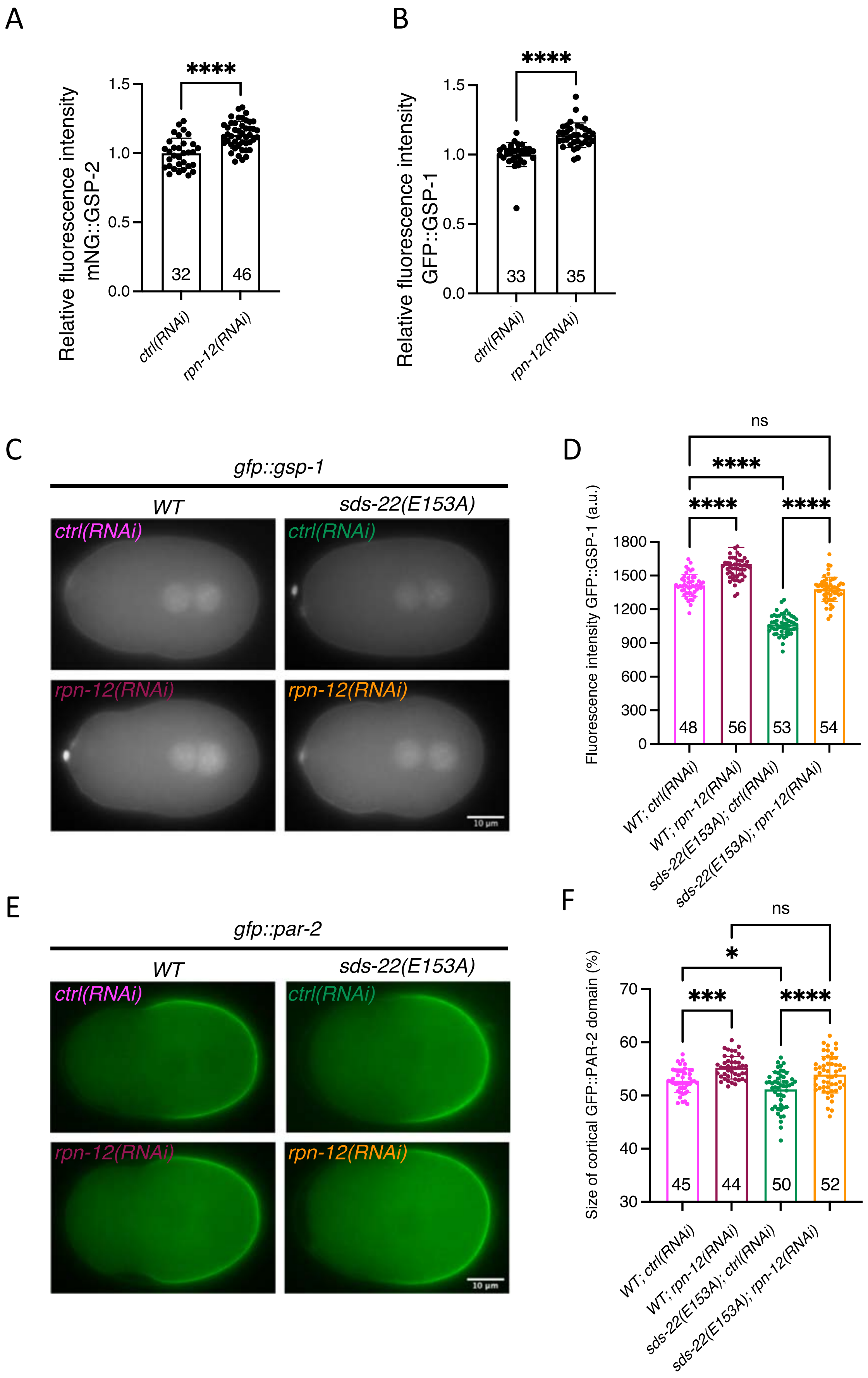
A
****
Relative fluorescence intensity mNG::GSP-2
1.5
1.0
0.5
0.0
32
46
ctrl(RNAi)
rpn-12(RNAi)
B
****
Relative fluorescence intensity GFP::GSP-1
1.5
1.0
0.5
0.0
33
35
ctrl(RNAi)
rpn-12(RNAi)
C
gfp::gsp-1
WT
sds-22(E153A)
ctrl(RNAi)
ctrl(RNAi)
rpn-12(RNAi)
rpn-12(RNAi)
10 µm
D
ns
****
****
****
Fluorescence intensity GFP::GSP-1 (a.u.)
1800
1500
1200
900
600
300
0
48
56
53
54
WT; ctrl(RNAi)
WT; rpn-12(RNAi)
sds-22(E153A); ctrl(RNAi)
sds-22(E153A); rpn-12(RNAi)
E
gfp::par-2
WT
sds-22(E153A)
ctrl(RNAi)
ctrl(RNAi)
rpn-12(RNAi)
rpn-12(RNAi)
10 µm
F
ns
*
***
****
Size of cortical GFP::PAR-2 domain (%)
70
60
50
40
30
45
44
50
52
WT; ctrl(RNAi)
WT; rpn-12(RNAi)
sds-22(E153A); ctrl(RNAi)
sds-22(E153A); rpn-12(RNAi)

Figure EV5. Depletion of RPN-12 increases GSP-1/-2 levels and PAR-2 domain length.

(**A**, **B**) Quantification of mNG::GSP-2 and GFP::GSP-1 in *ctrl(RNAi)* and *rpn-12(RNAi)*. $N = 2$. The *P* values were determined using two-tailed unpaired Student's *t* test. (**C**) Representative images of *gfp::gsp-1* and *gfp::gsp-1; sds-22(E153A)* embryos in *ctrl(RNAi)* and *rpn-12(RNAi)*. (**D**) Quantification of GFP::GSP-1 intensity levels. $N = 3$. (**E**) Representative images of *gfp::par-2* and *gfp::par-2; sds-22(E153A)* embryos in *ctrl(RNAi)* and *rpn-12(RNAi)*. (**F**) Quantification of the GFP::PAR-2 domain size at pronuclear meeting in live zygotes. $N = 3$. For all embryos, the scale bar is 10 μm, anterior is to the left and posterior to the right. Mean is shown and error bars indicate SD. Each dot represents the measurement of one embryo. The *P* values were determined using one-way ANOVA "Tukey's multiple comparisons test". Sample size (*n*) is indicated inside the bars in the graph. In all plots, ns$P > 0.05$, *$P < 0.05$, **$P < 0.01$, ***$P < 0.001$, ****$P < 0.0001$. Exact *P* values are provided in Dataset EV3. *n* number of embryos analyzed, *N* number of independent experiments.

© The Author(s)
